# An Automatic Movement Monitoring Method for Group-Housed Pigs

**DOI:** 10.3390/ani14202985

**Published:** 2024-10-16

**Authors:** Ziyuan Liang, Aijun Xu, Junhua Ye, Suyin Zhou, Xiaoxing Weng, Sian Bao

**Affiliations:** 1 School of Mathematics and Computer Science, Zhejiang Agriculture and Forestry University, Hangzhou 311300, China; hi@ziyuan.work (Z.L.); ajxu_zafu@163.com (A.X.); 2 School of Environmental and Resource Science, Zhejiang Agriculture and Forestry University, Hangzhou 311300, China; yejunhua2020@zafu.edu.cn; 3 Zhejiang Academy of Agricultural Machinery, Jinhua 321000, China; yuting11023@126.com; 4 Zhongrun Agriculture and Animal Husbandry Technology (Zhejiang) Co., Ltd., Jinhua 321000, China; sky354523482@163.com

**Keywords:** pig, movement monitoring, YOLOv8, agglomerative clustering, spatial moment

## Abstract

Continuous movement monitoring helps quickly identify pig abnormalities, enabling immediate action to enhance pig welfare. However, continuous and precise monitoring of daily pig movement on farms remains challenging. We present an approach to automatically and precisely monitor the movement of group-housed pigs. The instance segmentation model YOLOv8m-seg was applied to detect the presence of pigs. We then applied a spatial moment algorithm to quantitatively summarize each detected pig’s contour as a corresponding center point. The agglomerative clustering (AC) algorithm was subsequently used to gather the pig center points of a single frame into one point representing the group-housed pigs’ position, and the movement volume was obtained by calculating the displacements of the clustered group-housed pigs’ center points of consecutive frames. We employed the method to monitor the movement of group-housed pigs from April to July 2023; more than 1500 h of top-down pig videos were recorded by a surveillance camera. The F1 scores of the trained YOLOv8m-seg model during training were greater than 90% across most confidence levels, and the model achieved an mAP50-95 of 0.96. The AC algorithm performs with an average extraction time of less than 1 millisecond; this method can run efficiently on commodity hardware.

## Simple Summary

Pork production constitutes a significant portion of global meat production, with projections indicating a substantial increase in global pork consumption by 2032. Pig welfare is important not only for the pigs themselves but also for the volume of pork produced. To address the challenge of continuous and accurate daily monitoring of pig movement on farms, this paper further explores pig movement monitoring methods by combining image deep learning and unsupervised clustering algorithms.

## 1. Introduction

Pork is the second largest type of meat produced worldwide. Specifically, pork production reached 109 million tons in 2020, accounting for 33% of global meat production [1]. The OECD projected that global pork consumption will increase by 11% by 2032 [2]. The volume of pork production is closely related to pig welfare, and both farmers and consumers have become increasingly concerned with the animal living conditions present in the pig farming industry [3,4,5]. Uncomfortable and sick pigs exhibit abnormal behaviors, including a sharp decrease in daily movement. Therefore, monitoring pig movement is crucial in growing farming practices [6,7,8]. Continuous pig movement monitoring contributes to the further enhancement of swine welfare, and pig movement monitoring approaches are important in behavioral research.

Human observation and equipment-based methods are commonly used in pig movement monitoring. The former is limited since pigs are curious and use their noses to touch humans [9]. On the basis of the field observation, humans can influence the natural movement of pigs, resulting in them running or approaching humans. In addition, observational results inherently vary from person to person [10]. Methods employing equipment such as RGB cameras have been used in numerous pig research studies, and camera-based methods have become popular because of the convenience of data recording in precision livestock farming (PLF) [11]. Nasirahmadi et al. used a camera to achieve posture recognition for pigs with different body colors [12]. Hansen et al. used a camera to achieve facial recognition of pigs with different facial colors [13]. Thus, equipment-based methods are convenient, reliable, and sustainable for pig movement research.

Many experts have designed various approaches based on diverse equipment to monitor pig movement in pens. Pig movement monitoring equipment can be divided into invasive and non-invasive equipment. Pan et al. used an inertial measurement unit (IMU) device that attaches to a pig’s ear for pig behavior data collection and adopted deep learning analysis to monitor pig movement [14]. While an IMU device can be used to precisely record the activity information of a single pig, the breeding cost increases rapidly when every pig wears an IMU device in a scaled farming pen. Additionally, these invasive devices cause additional stress to pigs and thus are harmful to pig welfare [15]. In recent years, the use of non-invasive equipment has been popular in pig movement research to avoid frightening pigs during monitoring. Dong et al. deposited sensors around a sow cage to collect information on pig movement and proposed a deep learning algorithm to classify pig activity levels [16]. The sensors require custom designs and may present challenges in deployment convenience. Other efforts have begun to research the analysis of pig movement images or videos. Larsen et al. combined typical computer vision approaches and statistical methods to quantify piglet locomotor contours in pictures and analyzed pig movement patterns [17]. This method requires time to process every frame image manually. Wei et al. proposed a deep-learning-based method to detect pig movements and behaviors from a few consecutive frames [18]. Melfsen et al. used YOLOv7 to analyze pig activity and behavior patterns [19]. Ho et al. used deep learning to track piglets and successfully quantified their movement in short videos [20]. This method tracks piglets for only a few minutes, and the precision of pig tracking decreases over time. Ding et al. used improved YOLOv5 to quantify piglet movement in sow cages [21]. This method quantifies the movement level on the basis of a complete frame image, but it does not obtain the piglets’ position and is unable to determine the displacement of the frame images. Xu et al. used a deep learning detection model to detect pigs and recognized each pig’s location by computing the center position of its bounding box peaks [8]. However, pig location is quantified only at the box level in this way, and subtle movement might be ignored because pigs occasionally exhibit behaviors such as walking in place, during which small movements that do not change the position of the detected box occur. We summarize the current limitations according to the PLF trend and the proposed methods: (1) Methods that utilize traditional computer vision techniques to monitor pig movement have a low degree of automation. (2) Image processing methods can acquire the trajectories of moving pigs; however, these methods cannot be used to quantify group-housed pigs’ position and displacement. (3) Full-day tracking of individual pigs is difficult due to imperfect recognition accuracy, which results in the missing information of individual pigs in random frames; moreover, interactions between individual pig bodies can lead to confusion during the identification process, which leads to low precision in individual pig movement tracking and a lack of robustness in group pig monitoring. Therefore, developing an automatic, continuous and highly precise method for monitoring and quantifying the movement of group-housed pigs has considerable research value for the modern pig farming industry.

Object detection methods, such as the region-based convolutional neural network (R-CNN) model [22] and the YOLO model [23], can be utilized to identify various objects in image frames. This object detection task provides a mask of the generated bounding boxes after target detection. However, image segmentation methods outperform object detection because they can detect objects in images and accurately delineate their contours at the pixel level, enabling the separation of individual objects from the background and other objects in an image. Therefore, segmentation algorithms have been widely used in the field of pig husbandry. Tu et al. used the frame difference algorithm to subtract frame backgrounds from consecutive frames, creating segmented images of pigs in consecutive frames and graphically estimating pig weights by combining the segmented image pixels with live pig data [24]. However, the images generated via the background subtraction method contain a significant amount of abnormal pixel noise, which impacts the pig recognition results. Xu et al. used the GrabCut algorithm to process pig scene images and locate pig positions [7], but their processing of each image required substantial human intervention to meticulously adjust the parameters of the background image. Traditional computer vision algorithms also have difficulty segmenting pigs from complex backgrounds [25]. Instance segmentation is a revolutionary technique that combines object detection and semantic segmentation. YOLOv8-seg is a real-time instance segmentation algorithm extended from YOLOv8 [23], and its segmentation capacity is based on the YOLACT algorithm [26]. YOLACT achieves faster execution than mainstream instance segmentation algorithms (such as Mask R-CNN). We introduce the YOLOv8-seg model to detect the positions of group-housed pigs to improve the precision and level of automation of pig movement monitoring.

We developed an approach for continuously monitoring group-housed pigs via the following steps: (1) The deep learning model YOLOv8m-seg is used to automatically and precisely detect pigs in images and acquire points that consist of their body contours. (2) According to the pig contour points, the geometric spatial moment algorithm is used to quantitatively summarize the location of every pig as a single point, and the locations are separately determined on the basis of the detection results for each frame. (3) After every quantified pig’s location is obtained, the agglomerative clustering algorithm is applied to characterize the movement of the quantified pigs in terms of a single center point, and an improved Euclidean distance algorithm is used to increase the robustness of this method. We successfully deployed this automatic approach to obtain daily monitoring information on pig movement.

## 2. Experimental Materials and Methods

### 2.1. Pigsty Environment

**Figure 1:**
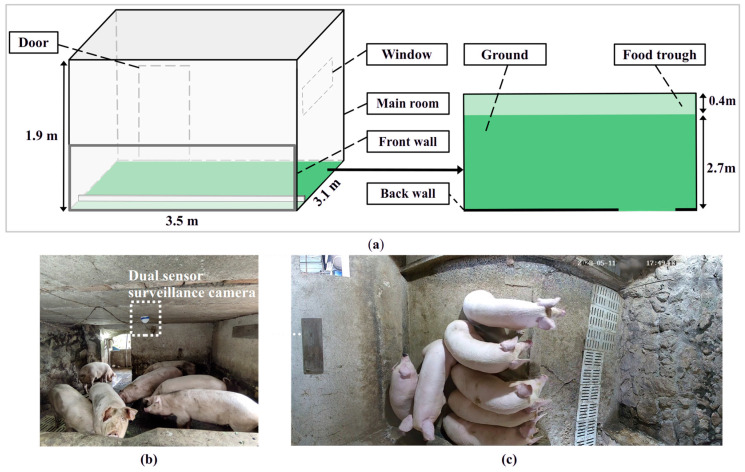
Experimental conditions: (**a**) draft of the pigpen; (**b**) installation position of the dual sensor surveillance camera; (**c**) top-down camera view of the pigsty floor.

In this research, the research pigpen, a pigpen in the countryside, was located in Hangzhou, China. The pigsty was constructed with stone and contained an open, wide environment, which helps keep the inside dry and clean and is convenient for breeding. The draft of this pigpen is shown in Figure 1a. The main pig room was 3.5 m × 3.1 m × 1.9 m (length × width × height) and included an open food trough (3.5 m × 0.4 m) set close to the front wall, a gutter embedded in the stone floor, and a full-day open window on the left wall that was left open for ventilation. A door in the back wall connects the main room to either a 3 m^2^ room or a second room with unrestricted access for the pigs to facilitate excretion and water intake. This study included 7 large white pigs, the ages of the pigs were between 100 and 120 days, and the average activity space per pig was greater than 1.8 m^2^. The farm owners usually fed them commercial feedstuffs mixed with chaff and fresh vegetables, and the frequency of feeding was twice a day. All the rooms were cleaned to prevent bacterial growth when pigs finished intake. In our research, we studied only the pig room (main room) area. The temperature of the pig room ranged from 22 °C to 30 °C, and the humidity ranged from 60 to 80%.

### 2.2. Equipment Preparation

In this research, we used a commercial surveillance camera to collect full-day videos of pig movement in the pigsty. A surveillance camera can provide high-quality and continuous records. Generally, a single-lens camera has a maximum focal length of 4 mm, which prevents data acquisition in the entire area of the pig room. A spherical camera can collect data over the entire field of view (FOV), but it generates frames with large distortions. To capture ideal images, we choose a wide dual-sensor surveillance camera (HIKVISION, model DS-2CD3346WDP2V2-L, Hangzhou, China) with a 2.8 mm focal length and 180-degree horizontal FOV. The camera automatically combines two sensors into one frame with a wider FOV; it provides up to 6-megapixel (3632 × 1632 pixels) resolution and 20 fps video recordings, and it has an automatic night vision function (Figure 1b). To acquire ground pig movement data, we installed the aforementioned wide dual-sensor surveillance camera in the center of the pigsty ceiling, adjusting its view to be top-down as much as possible. We previewed the FOV by using a network video recorder (NVR) to communicate with the camera and fine-tune the FOV of the camera to cover the entire active swine area in the pig room. The NVR saved 24/7 video files recorded by the camera to its inner HDD (Seagate Technology, 8 terabytes, 3.5 inch, Fremont, CA, USA).

### 2.3. Data Collection and Processing

After the experimental equipment was installed, the pigs in the room lived normally. We collected full-day camera-recorded video data of pigs from NVR. All videos used in the research were recorded from April to July 2023, and the duration of the videos was more than 1500 h. All the recorded daily videos were fragmented, and they were sorted and connected by day to provide coverage for the morning, afternoon, and night.

The pig prediction model is trained with a training dataset, and the quality of the collected dataset impacts model performance. Specifically, selecting training data and processing these data contribute to building a high-performance model. The hardware environment for processing the data and running the model was an I7-13700KF CPU-based computer with an RTX4090 (24 G VRAM) GPU with 32 GB of memory equipped with a 2 TB PCIE4.0 SSD for storage. The software environment used was Python version 3.11, and PyCharm software version 2024.2.1 was used for program development. We used the FFmpeg library in Python to extract frames from the recorded videos. Overall, the observed activity of the pigs in the room was low, with instances where the pigs remained motionless for several minutes. Each image was captured at intervals of 5 min from every daily video, after which frames of daily pig movement were obtained. The frame extraction process ensures that highly similar images are removed, thereby increasing the diversity of the dataset. The images in the dataset were selected from the first few and last few days of the recording period, and the middle parts of the videos were used for continuous movement analysis. Motion blur (pig suddenly moving fast and locomotor afterimages remaining in the frame), overexposure (sunbeams shining on the pig bodies and ground, leading to pig contours that cannot be distinguished), dark regions (pigs laying together after nightfall and times when the camera night vision function was not instantly activated so that pigs could not be recognized in frames by human observations), and similar images (group-housed pigs remaining in the same position anteroposterior for a few minutes) were manually excluded; these bad images obstructed the annotation process. Finally, 180 different pig scene images were obtained from the videos and were used in the subsequent dataset setup phase.

### 2.4. Dataset Establishment

We utilized Roboflow online software (https://roboflow.com (accessed on 25 January 2024)) [27] to annotate the above pig scene images. Instance segmentation labeling requires point-to-point connections of the object’s contour, each pig’s contour requires around 30 to 50 points to be labeled; on the basis of the contours of the group-housed pigs, we manually annotated approximately 20 thousand annotation points of pigs in this stage.

After annotating the data, we needed to modify the data before establishing the dataset. Moreover, increasing the number of images can improve model performance. Our annotated images were augmented through computer vision processing with the following strategies: flipping (horizontally flipping the image), hue alteration (between −25° and +25°), changes in the degree of exposure (between −30% and +30%), and the addition of image noise (up to 5% of the pixels). After finishing the data augmentation process on the basis of the 180 raw pig scene images, we acquired a total of 446 images for the dataset.

### 2.5. Movement Monitoring Method for Group-Housed Pigs

**Figure 2:**
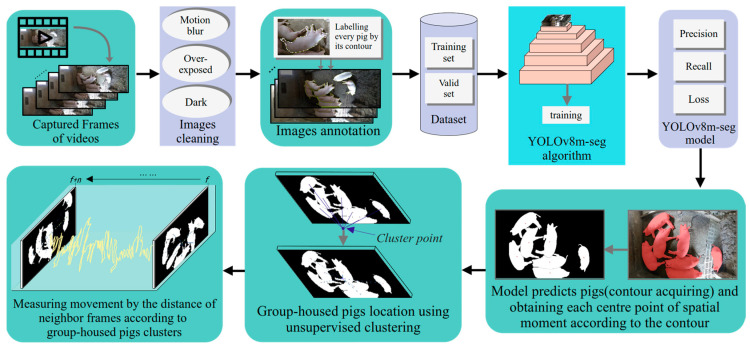
Designed workflow of the pig movement monitoring method.

The observation of a single pig does not reflect group status. For continuous monitoring of group-housed pig movement, we designed an effective approach (Figure 2). The generated dataset was used for model training and testing. First, we applied a high-speed artificial intelligence algorithm to train a pig prediction model. The model automatically detects pigs and outputs porcine contour coordinate information (Section 2.5.1). Second, after the results of different center calculation algorithms were compared, the spatial moment algorithm was used to quantify individual pigs, which means that pig contour coordinates were calculated at one center point that represents the pig location in the pigsty (Section 2.5.2). Third, we selected a fast computational clustering algorithm to gather all the center points of the pig contours, with a single cluster point representing the position of the group-housed pigs (Section 2.5.3). Finally, we calculated the displacement of the cluster points in adjacent frames to obtain the pig movement information (Section 2.5.4).

#### 2.5.1. Detection Model for Group-Housed Pigs

**Figure 3:**
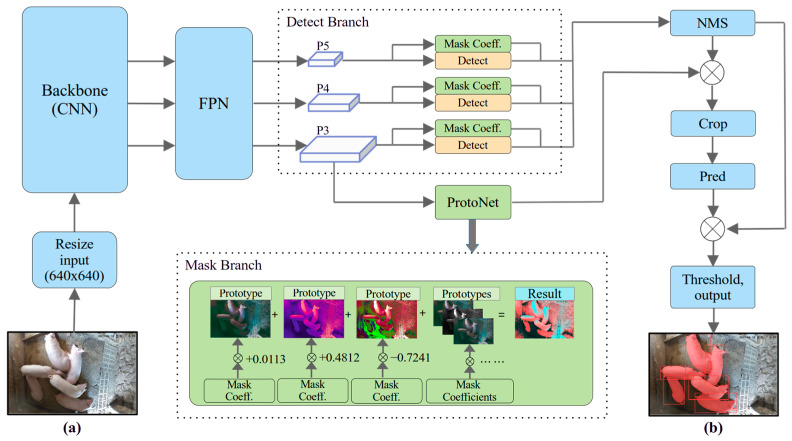
Model structure of YOLOv8-seg: the segmentation and detection tasks begin with the (**a**) original image and output an (**b**) image with a bounding box and segmentation contour.

The architecture of YOLOv8-seg was derived by modifying the design of YOLOv8 [23]. YOLOv8-seg is a novel instance segmentation algorithm; its structure is more advanced than that of previous mask R-CNN algorithms, and it has a high processing speed and improved detection efficiency. Figure 3 displays the core of the YOLOv8-seg model structure. Initially, the input image is resized to a default size of 640 × 640 × 3; then, the image is passed through a CSPDarknet53-based backbone network and feature pyramid network (FPN) for feature extraction [28,29]. The backbone consists of specific convolutional layers and pooling layers, which abstract image features into mathematical expressions, and the FPN addresses the differences in the scale information among the various detection layers, allowing for better detection of multiscale objects. The detection branch and mask branch concurrently operate after receiving the feature map. The YOLOv8-seg development team specifically altered the detection branch, a new module that can predict a batch of mask coefficients that cooperate with each detection head. Eighty bounding boxes with corresponding class labels were generated by the detection branch, and the non-maximum suppression (NMS) algorithm was applied to identify the optimal bounding box.

Moreover, an additional fully convolutional network (FCN) layer named the Proto module was used to output instance segmentation images in the mask branch of YOLOv8. The mask branch plays a crucial role in the segmentation process. This branch differentiates different instances in the same image and divides instances on the basis of pixel edges. The mask branch generates 32 mask coefficients by default, and the coefficients range from −1 to 1, with an accuracy of 0.0001; this approach is similar to the classification approaches used in detection branches. According to the mask coefficients, 32 alternative segmentation prototypes are output by processing the resized image. This mask branch receives a high-resolution feature map and preserves spatial details and semantic information. The feature map is generated by one convolutional layer through upscaling operations, the dimensions of the map are increased, and the map is passed to two additional convolutional layers to produce prototypical images. The instance segmentation images are obtained by assembling the generated mask prototypes. The mask multiplication formula is shown as follows:

(1) $$M=\sigma \left(PC^{T}\right)$$

Here, M represents the final mask image; P represents the pregenerated h × w × k matrix of mask prototypes; and C is an n × k matrix of mask coefficients, indicating the n instances that have passed through the NMS algorithm and score thresholding operation. In summary, processing the assembly stage of the designed instance segmentation algorithm is reliable and swift.

The segmentation branch produces the final masks. During training, the masks are cropped with the ground truth and detection bounding box separately, and the model performance is evaluated by comparing the area covered by the two boxes. When the model is used for prediction, the mask branch module crops the mask according to only the detected bounding box. The cropped images are passed through the instance prediction stage and threshold stage before the model outputs the prediction images. The model produces predicted images with contour points and confidence values at each point. These contour points contain coordinate information, which supports our next experiment involving pig location quantification.

#### 2.5.2. Location of the Pig Center Point

In the previous subsection, the pig contour points detected by the trained model were used for localization. In this subsection, we investigate and benchmark various center calculation algorithms. We then quantify the detected pig body contour as one point. The approach of using multiple points to track individual pigs in a group is insufficient because some pigs are resting, whereas others are moving; thus, this approach is insufficient for identifying group movement.

The previous pig detection phase involved determining each pig’s contour point information on the basis of an image. These contours include numerous coordinate points, and the pig locations can be determined graphically or analyzed statistically on the basis of these points. Because each pig’s contour includes many coordinate points, to locate the position of an individual pig while maintaining long-term statistical computational efficiency, all the contour points of a given pig are condensed into a single point via a mathematical center quantification method. Del Valle et al. calculated the center points of individual chickens in binary images via the coordinate average method [30]. The calculated center points fluctuate because of nonmoving animals’ activity, and the points are easily changed by different behaviors, which results in inaccurate locations. Hence, an algorithm that can determine the center point of a polygon instead of merely calculating the center of a simple geometric form enclosing that polygon is crucial for capturing pig movement.

**Figure 4:**
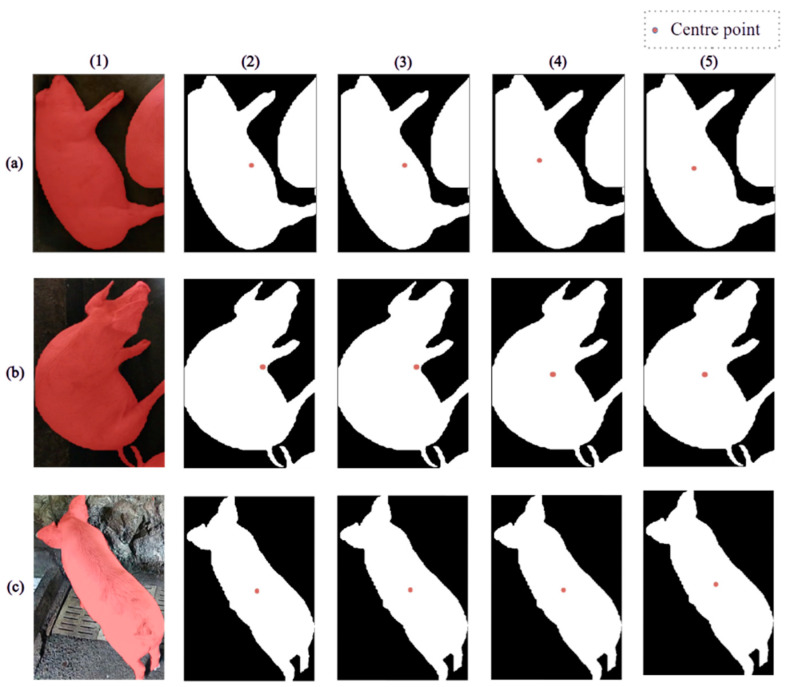
Distinguishing the center point of a predicted pig contour. The images in the columns are described as follows: (1) prediction image, (2) mean coordinate, (3) least squares, (4) signed area, and (5) spatial moment. The different pig behavior patterns depicted in each row are as follows: (**a**) lying, (**b**) sitting, and (**c**) standing.

Each pig contour consists of hundreds of points, and pig diurnal behavior patterns lead to contours with different shapes. The center point can be treated as a centroid because we use two-dimensional data. The algorithm for calculating the center point must be able to identify multiple forms. The pig ethogram varies with time; pigs can lie, sit, stand, stretch, or fold their legs naturally when lying [31]. Guo et al. used an algorithm that calculates the mean of the contour pixel coordinates to determine the centroid of a segmented pig in top-view images [32]. However, this algorithm yields an ideal result only when the pig is standing and the images are captured from a top view above the ground; in this case, identifying a pig in an image is similar to identifying an ellipse, which is helpful for calculating the centroid. In contrast, the contour of a lying pig consists of both an oval body and legs and thus resembles an anomalous polygon. There are many mathematical algorithms for calculating the center point of a polygon, including the mean coordinate, least squares, signed area, and spatial moment algorithms. Figure 4 shows the center points obtained via these algorithms for three pig postures: lying, sitting, and standing.

In the mean coordinate algorithm for calculating the center point of a polygon, the center point is obtained by averaging the coordinates of the peaks of the polygon. The center points of shapes with relatively few edges, such as circles, triangles, and rectangles, can be determined via this method. For a lying pig, the center point obtained in this way is closer to the side with the pig’s legs because the farthest coordinate points, namely the feet, have a higher weight in the calculation; thus, the calculated center point is not in the center of the pig body. Hence, this algorithm is inadequate for quantifying the locations of pigs with diverse contour patterns. Our second candidate approach is least squares regression. The computations needed for this approach are more complicated than those for the mean coordinate algorithm. This algorithm iteratively calculates and optimizes the center position to minimize the sum of the squared distances between each point and the center of the polygon. Using the least squares algorithm, we obtain a center point similar to that obtained with the mean coordinate method, differing only by a decimal; moreover, additional computations are needed. Thus, this method cannot find the ideal central location; instead, a weight-based center point algorithm is needed, as indicated by previous studies.

To obtain an appropriate weight-based algorithm, we can use the data in one area to quantitatively summarize the pig contour to a single center point. In the signed area approach, the polygon of interest is decomposed into a series of triangles; the area of each triangle is calculated, and a weighted average is then calculated to obtain the central coordinate. This algorithm represents the center point more accurately than the mean coordinate algorithm does; moreover, this algorithm is balanced by multiplying the centroids of the divided triangles rather than by statistically averaging the contour coordinates, and thus, the coordinates of the feet of a lying pig have less effect on the results. The center point acquired via the signed area approach is more closely aligned with the center of the pig’s body. However, this algorithm also has some shortcomings for the purpose of this research, as it does not properly account for small pig movements. When a pig that is lying down moves, even a small activity, such as turning the head or shifting a leg, can cause a large change in the center point, even if the pig remains still in consecutive frames.

To overcome the instability in the weight distribution along the pig contour, we can apply a moment-based algorithm. In this algorithm, the spatial moment is estimated in accordance with region-based geometric features obtained by extracting moment coefficients, and the position of the center point is then determined on the basis of the moment calculations. Even if part of the body appears to walk and the polygon changes slightly, the center point produced by the spatial moment algorithm will remain stable. Thus, the spatial moment algorithm yields an ideal result because of its weight-based foundation. The spatial moment algorithm is expressed as follows:

(2) $$m_{ji}=\sum_{x,y}\left(array\left(x,y\right)\times x^{j}\times y^{i}\right)$$

(3) $$\bar{x}=\frac{m_{10}}{m_{00}},\;\bar{y}=\frac{m_{01}}{m_{00}}$$

where $m_{ji}$ represents the spatial moment and j and i are the order of the moment. $m_{00}$, $m_{01}$, and $m_{10}$ denote the zeroth-order moment, first-order moment, and second-order moment, respectively. array(x, y) is the intensity for each pixel of the image. $\bar{x}$ and $\bar{y}$ represent the x-coordinate and y-coordinate of the spatial moment center point, respectively.

#### 2.5.3. Determination of the Position of the Group-Housed Pigs

The obtained center points were individually distributed in the scene images. Moreover, independent sporadic pigs are unideal movement monitoring targets. Furthermore, the greater the number of pigs is, the more computation time is needed. Separate points are not promising because they do not reflect the movement status of group-housed pigs. Thus, it is necessary to find a group-housed pig cluster point on the basis of all the pig center points. We collected scattered detected points to define a group of pigs via a clustering algorithm, and a series of variable cluster points represented different movement positions of the group of pigs. The continuous point data of the moving pigs were analyzed to distinguish temporal movement. Supervised clustering algorithms have been used in agricultural fields, such as image segmentation and crop data production [33,34]; however, these algorithms require additional human effort for data classification and dataset labeling. To process the mathematical statistics according to the center location of the group of pigs, considering that this is a two-dimensional coordinate calculation, a fast unsupervised clustering method can be prepared.

The candidate unsupervised clustering algorithms should be able to specify cluster points because only one point is needed in every frame. The candidate clustering algorithms include K-means++ [35], DBSCAN [36], mean shift [37], agglomerative clustering (AC) [38], and the Gaussian mixture model (GMM) [39]. These algorithms have been applied in various fields because of their specific characteristics. In general, the predicted coordinates of the pig group locations vary over time, and the clustering algorithms output coordinate points with different runtimes. Moreover, an excessively long group center calculation time may hinder the efficiency of the monitoring workflow. The mean shift algorithm iteratively searches for an appropriate center point on the basis of several candidates. The K-means++ algorithm is an optimization strategy that outperforms the original K-means algorithm in terms of iteration speed. The execution times of both algorithms depend on when the iteration process finishes. The runtime increases when dealing with clustering points that are difficult to discern. The GMM quantifies variables with multiple Gaussian distribution functions and was designed to improve convergence. Because the DBSCAN algorithm is density-based, it can rapidly compute the centers according to fewer expected clustering centers. The AC algorithm was designed as a bottom-up approach, with each point successively merged into clusters; this design helps decrease the computational complexity of the algorithm.

**Figure 5:**
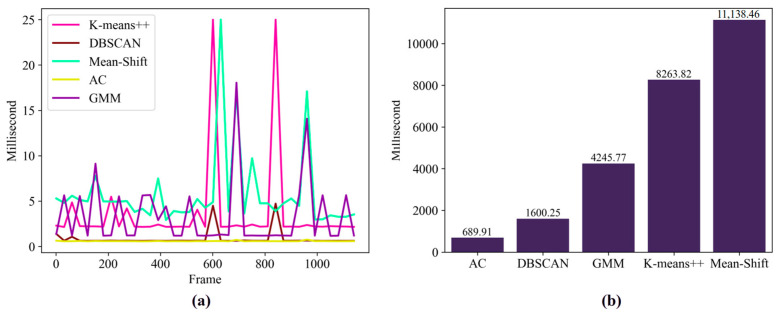
Running times of different algorithms based on the test video (1166 frames): (**a**) Time spent on each frame. (**b**) Total time spent in progress (average of 30 repetitions).

To select a suitable algorithm among the candidates, we obtained frame-by-frame pig center point data from a test video via the aforementioned moment method. The test video duration is 1 minute, and it was obtained from a 15 min video; it contains pigs lying down and continuously moving. The algorithms individually processed the data, and the runtime of each algorithm was recorded. The corresponding results are shown in Figure 5a. To display each algorithm’s runtime for each frame of the test video, a threshold was set to identify excessively long runtimes, which were then modified to 25 milliseconds when the figure was drawn. The data show that the AC and DBSCAN algorithms achieved fast performance; the remaining algorithms were unstable and occasionally had fast performance over a few frames. Figure 5b shows the total time consumed by the algorithms with the test video. The process was repeated 30 times in our experimental environment, and the results are presented as the average of the 30 data points. The bar graph displays the gradient disparity among the total time consumed by each clustering algorithm. The AC algorithm required the least time, whereas the mean shift algorithm required the longest time. We ultimately considered the AC algorithm to be a steady and fast clustering algorithm and therefore applied this algorithm in our experiments.

#### 2.5.4. Calculation of Group-Housed Pig Movement Distance

The movement trajectories of group-housed pigs in a frame were quantified at a given point in the previous clustering stage, during which the pig cluster point distances were calculated frame by frame to determine the movement of the pigs throughout the entire observation cycle. The final process involves obtaining the distance that the pigs moved by calculating the displacement of the cluster points in adjacent frames. The separation between two coordinate points is calculated by using the Euclidean distance. The pigs’ locations were predicted and quantified on the basis of the image pixels, and the obtained cluster coordinate points differed when the same image was input at different scales. To unify the statistical distance, we collected the absolute distance that the pigs moved on the basis of the Euclidean distance; the improved formula is as follows:

(4) $$D=\frac{\sqrt{{\left(x_{i}-x_{i-1}\right)}^{2}+{\left(y_{i}-y_{i-1}\right)}^{2}}}{\sqrt{H^{2}+W^{2}}}\times 100\%,$$

where D represents the absolute distance as a percentage; $x_{i}$ and $y_{i}$ are the *x*-axis and *y*-axis coordinate values of the current frame’s cluster point, respectively; $x_{i-1}$ and $y_{i-1}$ are the *x*-axis and *y*-axis coordinate values of the previous frame’s cluster point, respectively; and H and W represent the height and width of the image, respectively.

Two points can be used to determine the displacement, and according to the absolute distance moved by the group-housed pigs, images in the dataset with multiple types of scaling can still be evaluated. Thus, the final result is consistent even when a subset of the same dataset that has custom scaling is input. Without image scale limitations, the information was processed and plotted to evaluate the movement status of pigs via a robust approach.

## 3. Results

### 3.1. Training the Detection Model

YOLOv8-seg is based on deep learning technology and involves extensive iterative calculations. The annotated dataset was used to train YOLOv8m-seg (a version of YOLOv8-seg with fewer parameters). We set the number of epochs to 300, and the initial learning rate and final learning rate were 0.0001. To achieve the optimal model metric, the model was trained with pretraining weights. The images in the dataset were divided into a training set (399 images) and a validation set (47 images), and the training time was one hour in our experimental environment. After the model was trained, the model performance was assessed according to the evaluation criteria in every epoch. The precision (*P*) represents how precisely the trained model can predict the validation set. The recall (*R*) represents the ability of the training model to correctly identify all relevant instances within the given dataset. The formulas for *P* and *R* are as follows:

(5) $$P=\frac{TP}{\left(TP+FP\right)}$$

(6) $$R=\frac{TP}{\left(TP+FN\right)}$$

where true positives (TP) is the number of instances correctly predicted as positive, false positives (FP) is the number of instances that are actually negative but incorrectly predicted as positive, and false negatives (FN) is the number of instances that are actually positive but incorrectly predicted as negative.

*P* and *R* are model metrics only for evaluating the detection performance; thus, *P* and *R* were combined to create a new curve to evaluate the performance of our trained model. The *P*–*R* curve displays the performance of the trained model when classifying swine. Moreover, the F1 score combines *P* and *R* into a single assessment, balancing the two metrics to display their mean performance. The formula for the F1 score is shown below:

(7) $$F1score=2\times \frac{P\times R}{P+R}$$

The accuracy of the group-housed pig segmentation model was enhanced throughout the model training phase. The normal segmentation loss smoothly decreased as the number of epochs increased; otherwise, the trained model was overfit. The segmentation loss initially smooths in the 30th epoch during the training phase and validation phase. *P* and *R* quickly increased, then slightly fluctuated, and finally remained stable in the second half of the training stage. The trained model achieved a precision of 0.99 and an mAP50-95 of 0.96 [40]. The loss curve decreased, and the stable *P* and *R* values demonstrated that the model was adaptable to the data.

After the model was trained, the validation set was used to evaluate the model’s ability to detect unseen images. The objective confidence threshold for detection was set to 0.001. The *P* value increases with increasing confidence; the *P* value quickly exceeds 0.9 and then gradually approaches its maximum value before the confidence exceeds 0.6. The *R* value begins at the best value and starts to decrease slowly as the confidence increases to 0.6 and then decreases further when the confidence is close to 0.95. Finally, the F1 score curve shows that the performance that combines *P* and *R* is the highest; the F1 score is greater than 90% at most confidence levels and decreases to zero only when the confidence exceeds approximately 0.97.

### 3.2. Group-Housed Pig Movement Analysis

The best model was used to detect the location of the group-housed pigs. Images captured from the continuous videos were acquired from 13 May to 12 July and used for detection. None of these videos were included in the training dataset. The capture interval was 5 min, and approximately 15,000 images were obtained from the videos; the trained model achieved an average confidence of 96.2% after operating on the images.

**Figure 6:**
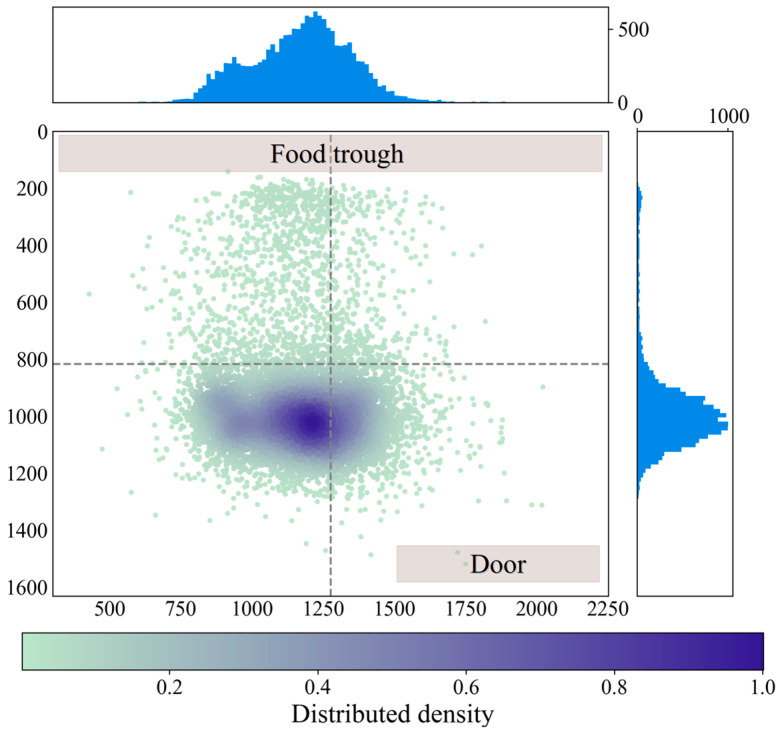
Complete distribution information of group pig positions. The information was obtained from 13 May to 8 July 2023, and every pig position was drawn at a given point.

All the detected positions from 13 May to 8 July are shown in Figure 6, which further shows the calculated positions of the detected pig herd. The x and y axes of Figure 6 represent the pixel coordinates of the image; the points are distributed across most of the pigpen area, reflecting that the pigs moved freely in this area. The color bar’s distributed density ranges from 0 to 1, such that the deep color area represents the pigs’ favorite resting place. The upper and right subfigures show histograms of the cumulative coordinate point distributions. The upper histogram shows smoother changes than the right histogram does. In the right histogram, higher values are distributed mainly in the lower part of the figure, and the magnitude of the change is greater than that in the upper histogram. During consecutive weeks of monitoring, pigs spent most of their time in the inner corner, close to the back wall of the pigpen.

**Figure 7:**
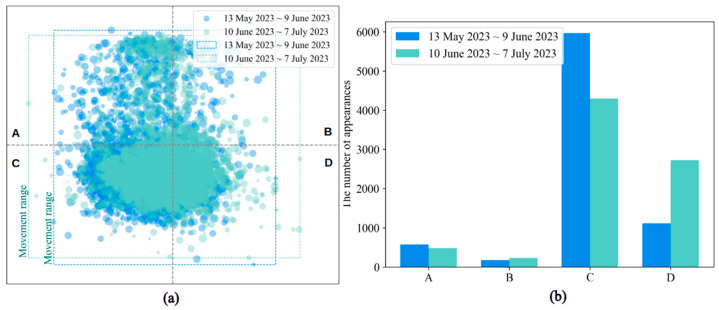
Changes in the position information over time: (**a**) Pigs positioned during two periods from 13 May to 9 June 2023 and 10 June to 8 July 2023. (**b**) The statistical variation in the number of pigs appearing in different regions during the two periods.

To further explore whether the pigs’ position distributions differed over time, we divided the detected positions into two periods, as shown in Figure 7. The first period was from 13 May to 9 June, and the second period was from 10 June to 8 July; each period lasted 28 days. There was no clear temporal variation in the movement habits of the pigs, and the regions with high-density distribution points in both periods indicated that pigs preferred to stay in the inner corners of the pigpen. According to these two periods, the movement area of the pigs in the pigpen was negligible over time.

Our research on pig movement positions revealed that the extent of the covered area was not related to temporal changes. To determine whether the coverage of the pig positions varied regionally with time, we counted the number of times each point fell into distinct regions during the two periods. Figure 7a is divided into four equal regions, denoted A, B, C, and D. Figure 7b illustrates the frequency of the pigs appearing in these four regions. According to the bar graph, pigs rarely appeared in Regions A and B. During the first period from 13 May to 9 June, the number of pigs that appeared in Region C was approximately 1.38 times greater than that during the second period from 10 June to 9 July. However, the number of pigs in Region D during the first period was 0.41 times greater than that during the second period. In terms of the number of pigs in different regions during the two periods, most pigs were in Region C, which is close to the inner wall.

### 3.3. Movement Distance of Group-Housed Pigs

**Figure 8:**
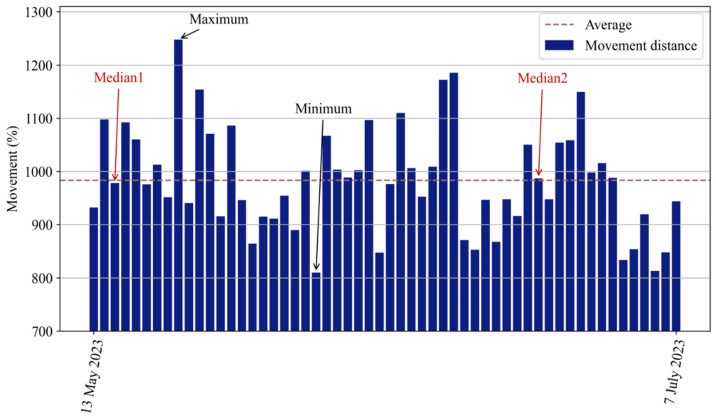
Daily summed movement distances of group-housed pigs from 13 May 2023 to 8 July 2023.

Figure 8 shows the summed volume of distance moved by the pigs each day. After all 56 days of movement were analyzed, the average movement distance was determined to be approximately 984%. The distance that the pigs moved each day differed from the average distance. The day with the maximum movement distance was 15 May, whereas the day with the minimum movement distance was 3 June. The longest movement distance was more than 1200% greater than the absolute distance, whereas the minimum movement distance was more than 800% greater than the absolute distance. According to the statistics of the daily movement distances, the median days were 15 May and 24 June. Ultimately, we determined the extent of the pigs’ daily movement distances.

**Figure 9:**
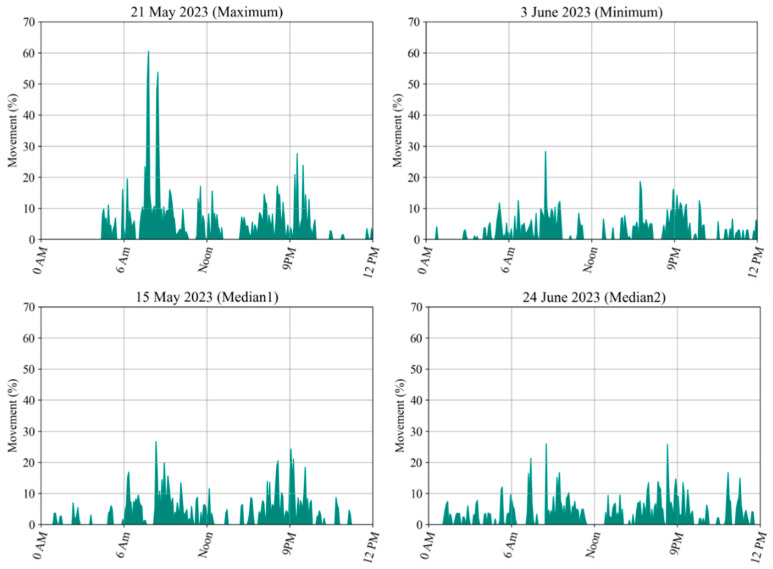
Movement characteristics of pigs in terms of days with the longest, shortest, and median movement distances; every subfigure starts at 0 a.m. and ends at 12 p.m.

To learn more about the differences in movement activities among the pigs, we selected four days that represent the maximum, minimum, and median movement distances separately for individual analysis (Figure 9). The movement distance on each day was determined from 0 a.m. to 12 p.m. On the day with the maximum movement distance, a unique large displacement was observed at 8 a.m., and the other movements on this day were comparable to those on the other days. However, the pigs did not move near midnight, and the pigs stayed in the same sleeping position at midnight as we observed in the original images. The movement details on these four days indicate that it is common for our pigs to decrease their movement at noon and even stop moving at this time. The movement of the pigs increased during the afternoon, and their walking behaviors usually continued into the night. We can observe in all four subfigures that two common movement peaks occurred at approximately 9 a.m. and 9 p.m.

## 4. Discussion

The movement of group-housed pigs was automatically monitored in this study, the proposed method can be used to monitor scale pig movement. In addition to tracking positions, future work will include monitoring lying down, drinking, and eating behaviors, and this comprehensive approach aims to improve pig welfare by detecting health issues and abnormal behaviors, aligning with modern practices in advanced pig farming. Understanding the movement position of pigs can help farmers determine whether changes in activity area ranges are due to dirty ground conditions. By monitoring the daily movement distances of group-housed pigs, farmers can identify decreased activity levels and promptly assess the health characteristics of the pigs. Movement information can reflect the living conditions of pigs, helping farmers improve the condition of farm animals in a timely manner [41,42].

The absolute Euclidean distance approach (Section 2.5.4) addresses inconsistencies in calculations due to the various image resolutions produced during the dataset generation stage. We compare this approach with recent related works (Table 1). To validate the model for continuous pig movement monitoring, our data cover a full day, whereas other approaches use specific periods of the day [17,20]. Those tracking algorithms provide real-time pig movement monitoring and reveal movement displacements, although long-term monitoring of this topic is difficult because of the increased tracking error rate caused by pig interactions, and a tracking algorithm is more beneficial for behavior detection rather than movement monitoring [18,20]. Ding et al. proposed a method that imports the spatial moment to increase the processing level of single pig location quantification into pixels, and the approach of quantifying pig movement levels on the basis of frames is prone to affecting the quality of images and is unable to reflect concrete pig movement positions [21]. On the basis of these improvements, we also further quantified movement displacement among group-housed pigs. Displacement information could help farmers learn whether and when pigs are active, but some recent approaches are not applicable [8,17].

**Figure 10:**
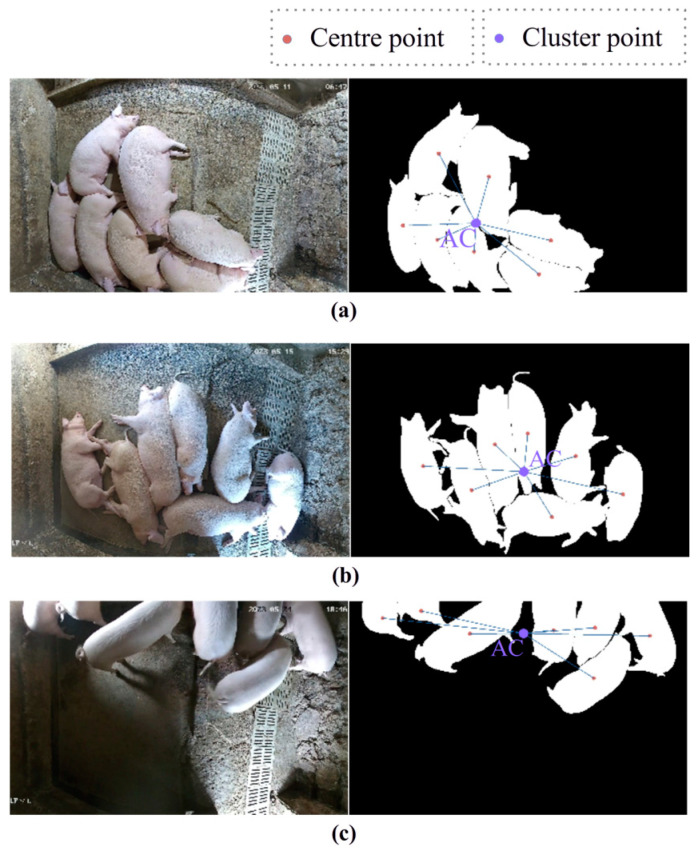
Various distribution locations of group-housed pigs: (**a**) pigs lying close to the corner; (**b**) pigs congregating near the door; (**c**) herd of pigs eating.

A review of our long-term automatic pig movement observations reveals that three common situations occur in the most active areas of the pigpen and can be identified on the basis of the pigs’ positions. Figure 6 shows the monthly locations of the pigs. Three main pig locations were analyzed, as shown in Figure 6. The three corresponding situations are shown in Figure 10. In the first situation, most of the pigs spend their time resting close to the corner, as shown in Figure 10a, which results in the greater density area observed in Figure 6. We propose that this area is densely populated because group-housed pigs tend to lie in cool and dark places. In the second situation, the pigs do not always remain in corners; rather, they sometimes gather near the doors, as shown in Figure 10b. After evaluating the temperature of the equipment in the pigpen, we determined that the temperature on the day when this activity was observed was lower than that on other days. The area near the pigpen’s door has more sunshine, and the pigs need to walk through this door to drink water. We checked the original images in which the pigs were close to the door and found that the pigs were resting in the area with the sun; the sunlight was strongest near the door. Future work can explore this situation in different pig pens. As shown in Figure 10c, in the third situation, the pigs eat at the food trough, causing a high-density area near the food trough. These pig movement habits, which are determined from quantified images, could help researchers better understand the behaviors of pigs.

Moreover, Figure 7a displays similar position ranges in the two periods, which indicates that the movement positions of the pigs were not significantly different across the two periods, suggesting that pigs’ movement habits remained similar even as the pigs grew. In other words, the position range has shown that pigs recognized their activity, which helps researchers understand pigs’ movement range and obtain an early warning. Only a few points are observed in the area near the food trough, indicating that pigs often do not move close to the food trough unless they are eating.

The movement distance of the group-housed pigs can reflect the level of activity within the pen; the summed movement distance of the pigs (Figure 8) shows less daily variation. We observed the daily movement of the pigs, and the daily data collected over a few days (Figure 9) revealed similarities in their movement distance patterns. These findings indicate that the main activity habits of pigs tend to be consistent across days, regardless of whether the daily total movement distance is greater or less than average. Moreover, the distances traveled by the monitored pigs varied at different times of day (Figure 10). Specifically, two distinct peaks, representing high movement levels, appeared at approximately 9 a.m. and 9 p.m. each day. Moreover, the observed nocturnal movement suggested that the peak movement at 9 p.m. may be attributed to the pigs searching for more comfortable positions for sleeping. Typically, the group-housed pigs exhibited low activity levels and relatively little movement, occasionally walking to find comfortable resting places. During certain monitoring periods, the pigs did not move any distance; at these times, the group of pigs was sleeping. A movement level of zero reflects the sleeping habits of pigs. When sleeping at night, pigs are unlikely to stay in the same position at all times; they might occasionally walk to another place, which might explain why some movement was identified at midnight. By analyzing the original images, we found that the group-housed pigs slept multiple times each day, usually during the late night, at noon, and during the afternoon. Further research could investigate how to combine such movement information with information about the farming environment, such as the level of carbon dioxide and the growth status of pigs, to address these differences.

## 5. Conclusions

To address the difficulty of precisely locating pigs and continuously monitoring pig movement, we developed a fast and precise method for automatically monitoring the movement of group-housed pigs. In this study, more than 15,000 images were extracted from the collected videos. A pig instance segmentation model based on YOLOv8m-seg was trained on a small subset of the collected images, and the trained model was subsequently used to segment the remaining images, with an average confidence of 96.2% and an instance segmentation mAP50-95 of 0.96. The spatial moment algorithm was used to quantitatively summarize the segmented contour of each pig to a single center point. The AC algorithm was subsequently applied to cluster the quantified pig’s center points in each frame into a single point to represent the overall location of the group-housed pigs. The proposed method was used to continuously quantify the movement of pigs on a farm from 13 May to 8 July 2023. During this period, the daily locations and movement distances of group-housed pigs in pens were automatically obtained. The average time needed to process each image was 218 milliseconds; compared to methods that require manual image processing, this method achieves automation. The proposed method can be used to automatically monitor the movement of pigs in a pigpen, thereby mitigating the time consumption issues faced when traditional monitoring methods are used. This method is suitable for monitoring the movement of group-housed pigs in any pigsty scenario covered by top-view cameras. Future work will decrease the computational requirements of the model to enable the deployment of the proposed method on microcomputer devices, which may facilitate the commercial use of this approach.

## Figures and Tables

**Table 1:** Comparison with recent approaches.

	[17]	[20]	[21]	[8]	[18]	Our Approach
Algorithm	CNN + LSTM	EfficientNet + LSTM	YOLOv5s + frame difference	YOLOv5s + XGBoost	GAM-YOLOv8	YOLOv8m-seg + spatial moment + AC
Data range	14:00–22:00 h 18:00–22:00 h	6:00–22:00 h	21:00–15:00 h	Full day	Daytime	Full day
Site	Farrowing pen	Farrowing pen	Farrowing pen	Pigpen	Pigpen	Pigpen
Object	Piglets	Piglets	Piglets	Pigs	Pigs	Pigs
Processing level	Bounding box	Bounding box	Frame	Bounding box	Bounding box	Pixel
Pig location	N/A	Yes	N/A	Yes	N/A	Yes
Displacement calculation	N/A	Yes	N/A	N/A	Yes	Yes
Performance	88% (Precision)	87.9% (mAP50 [40])	93.6% (Precision)	99% (mAP50)	96.2% (mAP50)	96% (mAP50-95)

Note: N/A represents this research did not address the work.

## Data Availability

The research data are available when required.
